# Diet Quality According to Mental Status and Associated Factors during Adulthood in Spain

**DOI:** 10.3390/nu13051727

**Published:** 2021-05-19

**Authors:** Jesús Cebrino, Silvia Portero de la Cruz

**Affiliations:** 1 Department of Preventive Medicine and Public Health, Faculty of Medicine, University of Seville, Avda. Doctor Fedriani, S/N, 41009 Seville, Spain; jcebrino@us.es; 2 Department of Nursing, Pharmacology and Physiotherapy, Faculty of Medicine and Nursing, University of Córdoba, Avda. Menéndez Pidal, S/N, 14071 Córdoba, Spain

**Keywords:** age groups, anxiety, depression, diet, mental health, national health and nutrition examination survey, population

## Abstract

Common mental disorders (CMD) are characterized by non-psychotic depressive symptoms, anxiety and somatic complaints, which affect the performance of daily activities. This study aimed to analyze prevalence of diet quality among adults with and without CMD from 2006 to 2017, to study the frequency of food consumption and diet quality according to mental status and age, and to determine which sociodemographic, lifestyle and health-related factors are associated with poor/moderate diet quality, according to mental status. A nationwide cross-sectional study was performed in adults with (*n* = 12,545) and without CMD (*n* = 48,079). The data were obtained from three Spanish National Health Surveys (2006, 2011/2012 and 2017). Two logistic regression analyses were used to identify factors associated with diet quality in people with and without CMD. Among those with CMD, the probability of having poor/moderate diet quality was significantly lower for overweight or obese people and those who took part in leisure-time physical activity. Among those without CMD, university graduates were less likely to have a poor/moderate diet quality. Good diet quality was observed more in older adults (≥65 years old) than in emerging (18–24 years old) or young adults (25–44 years old), regardless of mental status.

## 1. Introduction

Mental disorders constitute a rising public health concern throughout the world and are responsible for major social and economic issues affecting all age groups [1,2]. In the wide spectrum of mental illnesses, the most prevalent conditions are depressive and anxiety disorders, defined by the World Health Organization as common mental disorders (CMD) [3]. Although CMD are situated among the top 25 causes of the global burden of disease [4], they are in most cases, preventable and treatable [5,6,7]. Nevertheless, reducing the prevalence of CMD continues to pose a major challenge for health systems worldwide [8].

Many studies have examined the association between diet and depression, with only a limited exploration of anxiety and more severe mental illnesses. However, it is now clear that observational research nutritional psychiatry needs to be extended into this area [9,10,11]. Moreover, dietary intervention studies could not only provide far-reaching guidelines for the prevention and treatment of CMD in the future, but they could also further our current understanding of the associations between diet quality and CMD from an epidemiological viewpoint [12].

The influence of diet and nutrition on mental health and wellbeing is an emerging research avenue [13], and a poor diet quality has recently been revealed as a risk factor for CMD [14,15]. Over recent years, many studies have investigated the role of diet in the development of depression due to its influence on inflammatory pathways [16,17]. Here, extensive research has indicated that high levels of dietary inflammation are associated with increased risk of developing depression in different subgroups of the general population [16,17,18,19,20,21,22,23]. These findings highlight the need to considerer the promotion of an anti-inflammatory diet and healthy overall lifestyle as important elements in primary prevention strategies for CMD in the course of aging [23]. However, to confirm the role of a proinflammatory diet in depression, further prospective epidemiologic investigations are needed.

Optimal nutrition is critical to a person’s wellbeing and a healthy lifespan [24]. In this context, the scientific literature has shown that diet quality is worse in young people compared with older-age individuals [25,26,27]. For example, emerging (18–24 years old) and young adults (25–44 years old) usually have a poor quality diet, due to an excessive intake of energy-dense food and nutrient-poor meals, that include soft drinks, sweets and fast food, in comparison to older adults (≥65 years old) [28]. The inflammatory effects of a diet high in calories and saturated fat have been proposed as one mechanism that may have detrimental effects on brain health, including cognitive decline, hippocampal dysfunction, and damage to the blood-brain barrier [29]. This mechanism also presents a pathway through which poor diet could increase the risk of depression [30]. Some studies have reported that in emerging and young adults, levels of awareness about a balanced diet are low and, therefore, interventions are needed to provide opportunities for healthy dietary choices [31,32]. On the other hand, middle-aged (45–64 years old) and older adults are at important stages of life where events could impact on their diet quality, such as marital transitions, varying work-loads and children moving out of the home [33,34,35]. Here, promoting healthy dietary changes in these stages their lives and improving diet quality [36] could lead to an improved quality of life and help to reduce the burden of certain diseases [37]. Therefore, the key to managing non-communicable diseases related to diet and malnutrition is to take a life-course perspective, and steps must be taken to encourage a good quality diet in each stage of life by promoting a healthy diet [38].

A healthy dietary pattern is mainly characterized by a regular intake of fruit, vegetables, fish, seafood and whole grains [39]. The Mediterranean diet, which is associated with mental wellbeing [30,40], is a healthy dietary pattern characterized by a high intake of legumes, fruit and vegetables, nuts and seafood, a moderate alcohol intake, a low intake of red meat and saturated fat, and olive oil as the only or at least the main culinary fat source [41]. The anti-inflammatory nature of these foods may be the factor linked to a reduced prevalence of mental health conditions [42]. Most studies assess diet quality by the use of dietary quality indexes based on recommended dietary guidelines [43]. In the present study, we evaluated diet quality using the Spanish Healthy Eating Index (SHEI) [44], which conforms to the Spanish Society of Community Nutrition’s (SSCN) dietary guidelines [45].

In this novel study, we show the relationship between numerous different factors and diet quality in a large sample of adults with and without CMD in Spain surveyed in three waves (2006, 2011/2012, 2017) as part of the Spanish National Health Survey (SNHS). Therefore, the aims of this study are: (i) to analyze prevalence of diet quality among adults with and without CMD from 2006 to 2017, (ii) to study the frequency of food consumption and diet quality according to mental status and age, and (iii) to determine which sociodemographic, lifestyle and health-related factors are associated with poor/moderate diet quality, according to mental status.

## 2. Materials and Methods

### 2.1. Design, Data Source and Sample

A nationwide, cross-sectional study was carried out using individual data taken from three SNHS: 2006 [46], 2011/2012 [47] and 2017 [48].

The SNHS were conducted by the National Institute of Statistics and Spanish Minis-try of Health, Consumer Affairs and Social Welfare. These surveys were carried out on a representative sample of individuals via home-based personal interviews. The study sub-jects were selected by means of a probabilistic multistage sample, with census tracts as the first-stage units, family dwellings as the second-stage units and surveyable people present at home the third. SNHS collected data via in-home interviews which included specific questions on sociodemographic characteristics, mental health status and diet quality. Interviews were conducted by a suitably approved interviewer and supplemented in some cases by a follow-up telephone interview. Further details on the SNHS methodology can be obtained from the National Institute of Statistics [49,50,51].

From the SNHS database, we selected people aged ≥ 18 years old. From the initial 72,090 participants (SNHS 2006: *n* = 29,026; SNHS 2011/2012: *n* = 20,587; SNHS 2017: *n* = 22,477), we excluded 11,466 individuals who did not respond to or refused to answer the interview questions (SNHS 2006: *n* = 5162; SNHS 2011/2012: *n* = 3120; SNHS 2017: *n* = 3184). Therefore, the total sample of participants for the current study numbered 60,624 adults: 23,864 from SNHS 2006; 17,467 from SNHS 2011/2012; and 19,293 from SNHS 2017. For the nationwide, cross-sectional analysis, we included 48,079 adults without CMD (SNHS 2006: *n* = 18,657; SNHS 2011/2012: *n* = 13,721; SNHS 2017: *n* = 15,701) and 12,545 adults with CMD (SNHS 2006: *n* = 5207; SNHS 2011/2012: *n* = 3746; SNHS 2017: *n* = 3592).

### 2.2. Variables

#### 2.2.1. Diet Quality

The SHEI tool was used to study diet quality as a dependent variable [44]. The SHEI is an instrument designed to measure how well diets meet the recommendations of the SSCN dietary guidelines [45].

The SHEI questionnaire consists of ten food representative groups from the dietary guidelines, as follows: (a) daily consumption: (1) bread/grains, (2) vegetables (i.e., leafy vegetables, salads) (3) fruit (excluding juices) and (4) dairy products (yoghurt, cheese, milk). (b) Weekly consumption: (5) meat (lamb, pork, chicken) and (6) legumes. (c) Occasional food consumption: (7) cold meats and cuts, (8) sweets (pastries, cereals with sugar, biscuits, jams) and (9) soft drinks with sugar, and the last was (10) the variety of the diet, according to the SSCN recommendations for a healthy diet. The ten food items were worded in exactly the same way in the three SNHS. These items were divided into five response options, in the following order according to the frequency of food consumption: (i) never or hardly ever, (ii) <1 a week, (iii) 1–2 a week, (iv) ≥3 times a week, but not daily, (iv) and daily.

Each of the ten food groups was scored from 0 to 10 points (Supplementary Table S1), with the highest score in a food group denoting maximum compliance with the SSCN recommendations [45].

The overall score of the SHEI results was based exclusively on the frequency of food consumption rather than the quantity consumed and was calculated from the sum of the frequency of consumption of the ten representative food groups and ranged from 0 to 100 points: the ‘poor diet quality’ category corresponds with the lowest scores (SHEI score < 51 points), the ‘moderate diet quality’ category corresponds with the middle scores (SHEI score 51–80 points) and ‘good diet quality’ category corresponds with the highest scores (SHEI score > 80 points) [44].

#### 2.2.2. Mental Health

The Spanish version of the General Health Questionnaire (GHQ-12) [52,53,54] was used to detect CMD.

Questions were answered in a 4-point Likert-type response format, from 0 points (‘more than usual’) to 3 points (‘much less than usual’) and were scored on a bimodal response scale (0-0-1-1), according to the original GHQ method [50]. The total score there-fore ranges from 0 to 12 points. A cut-off of ≥3 points was chosen to estimate the pro-portion of participants without CMD (<3 points) and with CMD (≥3 points) [55].

#### 2.2.3. Sociodemographic Characteristics

Sociodemographic factors were analyzed as independent variables and were gathered by asking subjects the following questions: ‘What is your gender?’ (female, male), ‘What is your marital status?’ (never-married, married, widowed and separated/divorced), ‘What is your educational level?’ (without formal education, completed primary studies, completed secondary studies or professional training, and completed university studies), ‘What is your nationality?’ (Spanish, foreign), ‘What is the size of your town of your residence?’ (<10,000 inhabitants and ≥10,000 inhabitants), ‘How old are you?’ (the age was divided into four major life stages encompassing most of the human lifespan: emerging adults (18–24 years old) as proposed by Arnett [56], young adults (25–44 years old), middle-aged adults (45–64 years old) and older adults (≥65 years), as proposed by Erikson [57]) and ‘What is/was the occupation, profession or trade you perform or performed in your last job?’ (occupation was classified into the occupation social class categories reported by the Spanish Society of Epidemiology [58] as Classes I and II, Classes III and IV, Classes V and VI).

#### 2.2.4. Health-Related Variables

Information on health-related variables was collected through the following questions: ‘Can you tell me if you currently smoke?’ (yes, no), ‘Have you consumed any alcoholic drinks in the last twelve months?’ (yes, no). In addition, self-perceived health status was assessed with the following question: ‘What is your health status?’, to which the participants could answer: very good, good, fair, poor or very poor.

Finally, body mass index (BMI) was based on self-reported weight and height and was classified into four categories, following the World Health Organization [59]: underweight (BMI < 18.50 kg/m^2^), normal-weight (BMI ranging between 18.50 and 24.99 kg/m^2^), overweight (BMI ranging between 25.00 and 29.99 kg/m^2^) and obese (BMI ≥ 30 kg/m^2^).

#### 2.2.5. Lifestyle Variables

The SNHS measured lifestyle variables on the basis of the following questions: ‘Do you take part in any physical activity in your work or your main activity?’ (yes, no) and ‘Do you engage in any physical activity during your leisure time?’ (yes, no).

### 2.3. Ethical Aspects

Data from the three SNHS (2006, 2011/2012 and 2017) are available to the public and are stored in anonymized microdata [46,47,48]; as a result, no special permits were required for their use. According to Spanish law, the approval of an Ethics Committee was not required. This data is shown in the Supplementary File.

### 2.4. Statistical Analysis

Statistical methods used in data analysis were descriptive statistics, inferential statistics and regression technique. Descriptive statistics were presented with a count (n/%). We performed the Kolmogorov–Smirnov normality test before any comparison of quantitative variables between the groups. Inferential statistics used were the Chi-Square test, or Fisher’s exact test if the number of expected frequencies was >5 between qualitative variables; Student’s *t*-test to compare the means between two independent groups as a parametric test, and the Mann–Whitney U test as a non-parametric test. The kind of regression technique used was logistic regression. In particular, two binary logistic regressions in subjects with and without CMD were performed to identify the factors related to diet quality in each group. For the purpose of the analysis, the diet quality was recoded as a categorical variable into ‘good diet quality’ (if the SHEI score was above than 80 points) and ‘poor diet quality/moderate diet’ (if the SHEI score was below or equal to 80 points). The significant variables (*p* < 0.05) obtained in each univariate analysis were modelled in binary logistic regressions. The goodness of fit was corroborated with the Hosmer-Lemeshow test. The Wald statistic was used as a contrasting statistic. In addition, the crude and adjusted odds ratios (OR) were calculated with 95% confidence intervals. The presence of confounding and interaction were examined. The hypothesis tests were two-tailed and statistical significance was fixed at an alpha error of below 5%. The weighting coefficients included in the SNHS were applied in all the analyses to ensure representativeness. For the statistical analysis, the IBM SPSS Statistics version 25 program (IBM Corp, Armonk, NY, USA), licensed to the University of Seville (Spain) was used.

## 3. Results

### 3.1. Characteristics of Participants

A total of 60,624 adults (48,079 people without CMD and 12,545 people with CMD) participated in the present study. Females were much more likely to be represented among those without CMD (75.12%) than those with CMD (24.88%). Participants who were active during their leisure time were much more likely to be represented among those without CMD (83.04%) than those with CMD (16.96%). In contrast, adults who perceived ‘poor’ their health status were much more likely to be represented among those with CMD (56.52%) than those without CMD (43.48%). Other sociodemographic, lifestyle and health-related factors are shown in Table 1.

### 3.2. Prevalence of Diet Quality in SNHS 2006, SNHS 2011/2012 and SNHS 2017

Following the year of the survey (2006, 2011/2012 and 2017), no significant trend in diet quality was observed in participants with or without CMD.

The prevalence of moderate diet quality among people with CMD was 63.82% in 2006, 50.96% in 2011/2012 and 67.01% in 2017 (*p* = 0.85). Similarly, the percentage of people without CMD whose diet quality was moderate was 65.18% in 2006, 56.17% in 2011/2012 and 70.13% in 2017 (*p* = 0.74). On the other hand, the prevalence of good diet quality in individuals with CMD was 32.76% in 2006, 46.53% in 2011/2012 and 30.71% in 2017 (*p* = 0.89) and in people without CMD was 31.47% in 2006, 42.15% in 2011/2012 and 28.13% in 2017 (*p* = 0.82). Finally, the prevalence of poor diet quality in people with CMD was 3.42% in 2006, 2.51% in 2011/2012 and 2.28% in 2017 (*p* = 0.25) while in participants without CMD it was 3.35% in 2006, 1.68% in 2011/2012 and 1.74% in 2017 (*p* = 0.39).

### 3.3. Frequency of Food Consumption and Diet Quality According to Mental Status

Table 2 shows the frequency of food consumption and diet quality according to mental status among adults from all three survey periods (2006, 2011/2012 and 2017). A higher frequency of daily consumption of certain food groups was observed among people without CMD (bread/grains: 87.23% vs. 85.39% *p* < 0.001; fruit: 68.46% vs. 67.63% *p* < 0.001; dairy products: 87.20% vs. 86.24% *p* < 0.001), but not in vegetables (44.78% vs. 47.12% *p* < 0.001).

As regards weekly consumption, 1–2 weekly consumption of meat was lower among individuals without CMD (27.22% vs. 31.42% *p* < 0.001), while for legumes, it was higher among individuals without CMD (67.01% vs. 57.66% *p* < 0.001).

As for occasional food consumption, the consumption ‘never or hardly ever’ of cold meats and cuts, sweets, soft drinks with sugar consumption was lower among participants without CMD (13.13% vs. 19.03% *p* < 0.001; 17.01% vs. 20.93% *p* < 0.001; 48.92% vs. 55.36% *p* < 0.001, respectively).

Furthermore, a moderate diet quality was more prevalent in individuals without CMD (64.22% vs. 60.89% *p* < 0.001).

The relationships between overall diet quality score, food group scores and presence/absence of CMD are shown in Table 3. People without CMD meet more food-based dietary guidelines of the SSCN in relation to bread/grains, fruit, dairy products and legumes consumptions in comparison with people with CMD. In contrast, people with CMD meet more food-base dietary guidelines of the SSCN in relation to cold meats and cuts, sweets and soft drinks with sugar consumption in comparison with people without CMD. In total, the overall diet quality was better in people with CMD.

### 3.4. Comparison of the Frequency of Food Consumption and Diet Quality across Age Groups within Individuals with CMD

**Figure 1:**
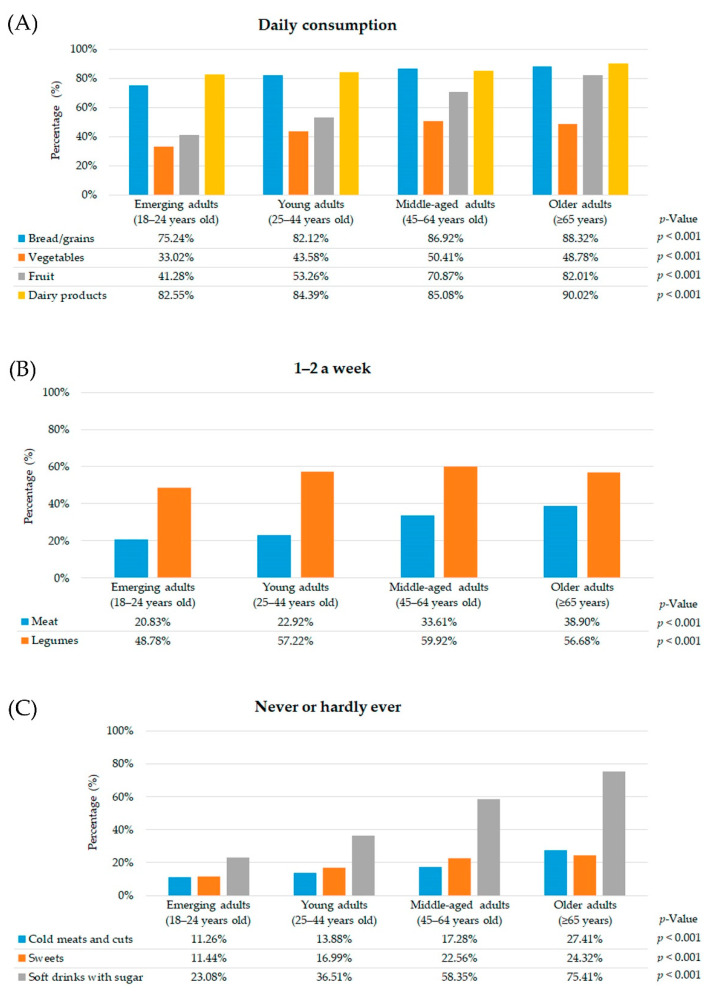
Frequency of food consumption according to age groups of participants with common mental disorders (*n* = 12,545) (SNHS 2006, SNHS 2011/2012 and SNHS 2017) ((**A**) for daily consumption, (**B**) for 1–2 a week, (**C**) for never or hardly ever).

Older adults with CMD had a higher prevalence of daily consumption of bread/grains, fruit and dairy products compared to other age groups. Nonetheless, vegetable consumption was higher among middle-aged adults than other age groups (Figure 1A).

In relation to weekly consumption, older adults with CMD had a significantly higher 1–2 weekly consumption of meat than other age groups. Meanwhile, the consumption of legumes 1–2 a week was more frequent in middle-aged adults with CMD compared to other age groups (Figure 1B).

As far as occasional food consumption is concerned, the prevalence of older adults with CMD who never or hardly ever consumed cold meats and cuts, sweets and soft drinks with sugar was higher than other age groups (Figure 1C).

Moreover, older adults with CMD had a significantly better diet quality in comparison with other age groups (emerging adults: 8.63%, young adults: 21.55%, middle-aged adults: 39.55%, and older adults: 51.20%; *p* < 0.001). In contrast, emerging adults had a higher moderate diet quality (emerging adults: 78.05%, young adults: 73.02%, middle-aged adults: 59.08%, older adults: 48.38%; *p* < 0.001) and poor diet quality (emerging adults: 13.32%, young adults: 5.43%, middle-aged adults: 1.37%, older adults: 0.42%; *p* < 0.001).

### 3.5. Comparison of the Frequency of Food Consumption and Diet Quality across Age Groups within Individuals without CMD

**Figure 2:**
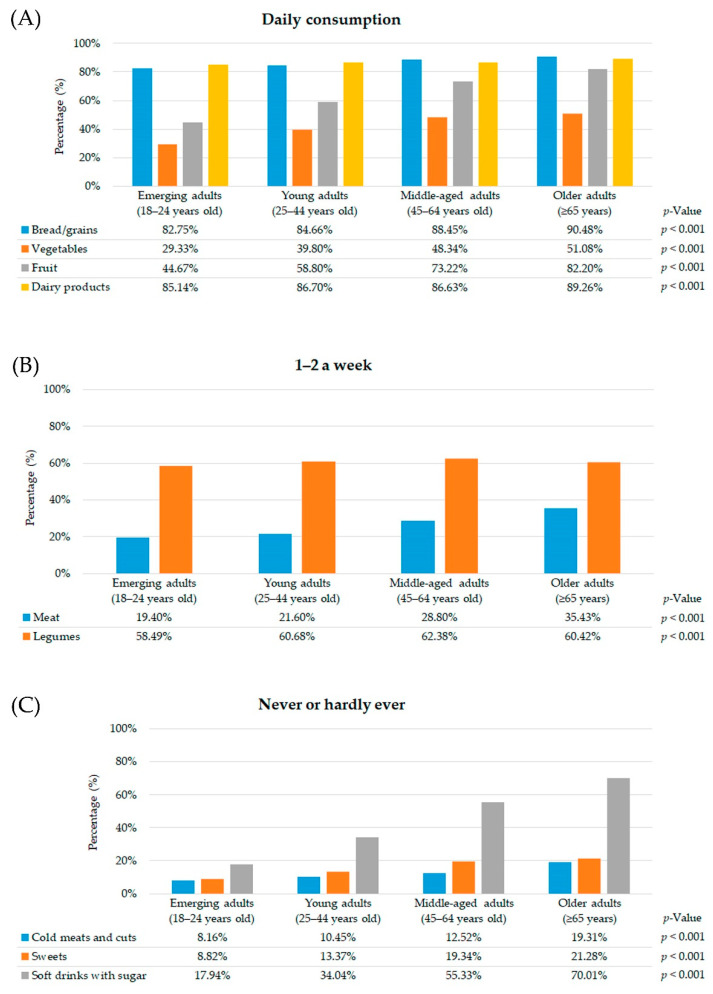
Frequency of food consumption according to age groups of participants without common mental disorders (*n* = 48,079) (SNHS 2006, SNHS 2011/2012 and SNHS 2017) ((**A**) for daily consumption, (**B**) for 1–2 a week, (**C**) for never or hardly ever).

Among participants without CMD, older adults met the existing dietary guidelines in terms of the daily consumption of bread/grains, vegetables, fruit and dairy products compared with the other age groups (Figure 2A).

As regards weekly consumption, the prevalence of 1–2 weekly consumption units of meat was higher among older adults without CMD. Nevertheless, middle-aged adults had a significantly higher 1–2 weekly consumption of legumes (Figure 2B).

In relation to occasional food consumption, the prevalence of older adults without CMD who never or hardly ever reported a daily consumption of cold meats and cuts, sweets and soft drinks with sugar was higher in comparison with other age groups (Figure 2C).

As for diet quality, moderate diet quality was higher in emerging adults without CMD (emerging adults: 78.62%, young adults: 75.07%, middle-aged adults: 61.29%, older adults: 48.42%; *p* < 0.001) than other age groups, as was poor diet quality (emerging adults: 10.38%, young adults: 3.67%, middle-aged adults: 0.98%, older adults: 0.30%; *p* < 0.001). In contrast, good diet quality was more prevalent in older adults without CMD (emerging adults: 11.00%, young adults: 21.26%, middle-aged adults: 37.73%, older adults: 51.28%; *p* < 0.001).

### 3.6. Comparison of the Frequency of Food Consumption and Diet Quality across Age Group in Individuals with and without CMD

As can be seen in Table 4, a higher frequency of daily consumption of bread/grains was observed among emerging and young adults without CMD in comparison with those with CMD. Moreover, a higher frequency of daily consumption of bread/grains was observed among middle-aged and older adults with CMD compared to those without CMD.

As regards weekly consumption, the consumption of meat was higher among middle-aged and older individuals with CMD in comparison with those without CMD. Similarly, 1–2 weekly consumption of legumes was higher among emerging and young adults without CMD compared to those with CMD.

As for occasional food consumption, the consumption ‘never or hardly ever’ of soft drinks with sugar consumption was higher among emerging and young adults without CMD in comparison with those with CMD. In contrast, the consumption ‘never or hardly ever’ of soft drinks with sugar was more prevalent in older adults with CMD compared to those without CMD.

In relation to diet quality, the prevalence of good diet quality among older adults with CMD was higher than those without CMD. On the other hand, the prevalence of poor diet quality among emerging adults without CMD was higher than those with CMD.

### 3.7. Association between Sociodemographic Characteristics, Lifestyle Behavior, Health-Related Variables and Diet Quality According to Mental Status

Among participants with CMD (Table 5), the adjusted logistic regression model indicated that the likelihood of having a poor or moderate diet quality was greater in individuals without formal education (OR = 1.24, 95% CI 1.09–1.40) and primary education (OR = 1.17, 95% CI 1.06–1.29). In contrast, lower rates of poor or moderate diet quality were linked to overweight (OR = 0.83, 95% CI 0.76–0.91) and obese (OR = 0.84, 95% CI 0.76–0.94) status, and those who took part in leisure-time physical activity (OR = 0.78, 95% CI 0.72–0.84).

The adjusted logistic regression model in Table 6 shows that the probability of having a poor or moderate diet quality was lower in people with university studies (OR = 0.90, 95% CI 0.84–0.95), and those belonging to Social Classes I and II (OR = 0.89, 95% CI 0.84–0.94). In contrast, higher rates of poor or moderate diet quality were linked to Social Classes V and VI (OR = 1.08, 95% CI 1.03–1.13), underweight status (OR = 1.47, 95% CI 1.23–1.77), people who had consumed alcohol (OR = 1.04, 95% CI 1.01–1.09), and those who took part in no leisure-time physical activity (OR = 1.47, 95% CI 1.40–1.53). Furthermore, the probability of having a poor or moderate diet quality was greater when the perceived health was fair (OR = 0.93, 95% CI 0.88–0.98) or very poor (OR = 0.80, 95% CI 0.64–0.99).

## 4. Discussion

### 4.1. Main Findings

The present study is unique in that it shows the relationship between a large number of characteristics and diet quality in a large sample of adults with and without CMD living in Spain, from a survey conducted in three waves (2006, 2011/2012 and 2017).

In our study, the prevalence of diet quality in need of improvement among people with and without CMD was higher in 2017 than in 2011/12. This may be due to the fact that in recent years, food habits and consumption in Spain are moving away from the traditional Mediterranean diet towards an increasingly “westernized” diet [60]. The Spanish diet is becoming saltier and sweeter due to the incorporation of more highly processed foods and changes in dietary habits. Specifically, the report on food consumption in Spain 2017 [61], conducted by the Ministry of Agriculture, Fisheries and Food shows that the prevalence of processed food consumption increased by 2.2% from 2011/12 to 2017. In addition, since 2015, there has been a reduction in the number of households with children and in the average family size. According to a previous study [62], people living alone are less likely to follow a varied diet and have lower fruit, vegetable, and fish consumption than those living with others.

There is a broad consensus in the scientific literature regarding the association be-tween the adherence to a poor diet quality and the presence of CMD [63,64,65,66]. The lack of energy or enthusiasm for preparing or enjoying food, as well as appetite loss, may influence diet quality among people with CMD symptoms [41,67]. Furthermore, emerging evidence suggests that diet may influence the onset of mood disorders and specifically depression. For instance, recent systematic reviews have demonstrated associations between measurements of diet quality and the probability and risk of depression [15,63]. Thus, diet may impact on in mental health via several pathways, including those related to oxidative stress, inflammation, and mitochondrial dysfunction, which are disrupted in people with mental disorders [68], and unhealthy diets contain certain compounds that may negatively affect these pathways. For example, elements commonly found in processed foods such as saturated fatty acids, artificial sweeteners, and emulsifiers may alter the gut microbiome and activate inflammatory pathways [43], which is associated with a significantly higher incidence of depressive symptoms, even among those without diagnosed mental disorders [17,20,69].

Our findings show that the prevalence of a moderate diet quality was higher in individuals without CMD. However, previous studies have pointed out that a history of CMD may stimulate these subjects to improve their diet quality in the long term [65,67].

Emerging adulthood (individuals aged 18 to 24 years old) is characterized by a continuing process of self-definition and increasing autonomy and it is a time when major role transitions take place [56,70,71]. Emerging adults tend to have a poor quality diet, often accompanied by an excessive intake of soft drinks with sugar and fast food [72]. Similarly, young adulthood is a period when healthy patterns are established, such as a good quality diet, which will carry over into later adulthood.

In our analysis, both emerging and young adults obtained poor diet quality index scores, regardless of their mental status. It is well-known that young people have an excessive intake of soft drinks with sugar, cold meats and cuts and sweets, while their intake of vegetables and fruit is lower in comparison with older age groups [73,74,75,76,77]. In fact, our findings showed that ‘never or hardly ever’ consuming soft drinks, sweets and cold meats and cuts was higher as age increased among people with CMD in comparison with those without CMD, except in older adults. Several possible mechanisms linking food with sugar intake and anxiety/depression symptoms are assumed, including inflammation markers [78]. Previous studies reported other influences on an unhealthy diet such as lack of motivation, time constraints and cost [79,80,81]. Meanwhile, a few intervention studies have correlated improvements in mental health status with an increase in fruit and vegetable intake [82,83,84,85]. For example, Mujcic and Oswald [86] showed that fruit and vegetables consumption increased happiness, wellbeing and life satisfaction. Moreover, middle-aged and especially older adults had a better diet quality in comparison with the two first life stages, although other studies in these advanced age groups with depressive symptoms found a poor diet quality [87,88], perhaps influenced by social factors, such as marital status and social contacts [89]. Our results show that a higher frequency of daily consumption of bread/grains, vegetables or fruit was observed among middle-aged and older adults with CMD in comparison with those without CMD. A higher intake of dietary fiber helps the nervous system, which has a beneficial influence on mental health [90]. Other food groups, such as legumes, contain B vitamins, magnesium, folic acid and potassium, among others, which could act as protection against the risk of psychological disorders [91]. Our findings show that 1–2 weekly consumption of legumes was higher among emerging and young adults without CMD compared to those with CMD, and among middle-aged and older adults with CMD in comparison with those without CMD. A randomized clinical trial suggests that a dietary rich in legume and nuts had beneficial effects on depression [92]. As regards the 1–2 weekly consumption of meat, it was higher among middle-aged and older adults with CMD, in line with another study [93].

Educational level may be one of most important social factors explaining differences in food habits [94,95,96]. Here, it was found that the probability of having a poor or moderate diet quality was greater in adults with CMD with a lower educational level compared with those without CMD with higher education. This is not the first time that a higher educational level has been associated with good dietary habits [97,98,99], although some studies have failed to find any association [100] or even found an inverse link [101,102], probably due to people with a higher educational level having access to better knowledge about food [103,104]. In addition, some studies have shown that a favorable attitude towards a healthy diet was associated with a higher educational level [105,106,107]. Our results contrast with another study, which found that university students whose diet was in need of improvement or had a poor diet had twice or triple the risk of CMD, respectively, than those who had a healthy diet [108].

Several studies have found an association between poor or moderate diet quality; the latter indicates a medium adherence to the recommendations proposed by the Spanish Society of Community Nutrition [45] and high BMI [109,110,111]. Nevertheless, our results have shown that overweight and obese were protective factors for poor or moderate diet quality in adults with CMD. Recently, it has also been shown that obese and overweight adults are motivated to lose weight by taking up a healthier lifestyle, such as healthy eating or doing physical activity [112]. Moreover, the sensory pleasure derived from eating palatable meals could reduce stress and produce a positive mood [113]. In contrast, being underweight was a risk factor for poor or moderate diet quality in adults without CMD, in line with other studies [114,115,116], probably due to a reduced intake of nutritious food [117].

As regards lifestyle behavior, the multivariate analysis showed a link between lower rates of poor or moderate diet quality and those who engaged in physical activity during their leisure time among adults with CMD. Moreover, not engaging in physical activity was a risk factor for poor or moderate diet quality in adults without CMD. It is well-documented that leisure-time physical activity has considerable mental health benefits throughout a patient’s lifespan [118,119,120,121]. In fact, engaging in leisure-time physical activity is negatively associated with CMD symptoms [122]. In addition, several studies have found many cases of individuals who had a good diet quality, and took part in healthy behaviors associated with engaging in physical activity and smoking less [123,124,125].

### 4.2. Strengths and Limitations

This study has certain limitations. First, the present study is a cross-sectional design, so it is currently not possible to assign causality. Second, diet quality was based exclusively on the frequency of food consumption and not on the quantity of consumption; this does not allow us to judge if the *p*-values given for the small percentages obtained for CMD versus non-CMD are clinically significant despite the fact that the sample size is very large. Third, mental status was constructed from self-reported questionnaires, and the responses could therefore have been biased by social desirability and/or memory. Fourth, there was a lack of data on institutionalized populations, since the personal interviews were conducted at the participants’ homes. Fifth, the BMI data were taken from the participants’ own responses about their height and weight in the self-reported surveys. Finally, the SNHS surveys were carried out with different samples, which means that the different characteristics of the participants could have affected our results.

On the other hand, one strength of the present study is that data from the SNHS surveys have been obtained using carefully planned methodology, including sampling, well-designed forms, preparation of the survey participants, supervision of the survey and filtering of the data, all of which guarantee a representative sample of the population and enable comparisons to be made. Moreover, the validity of the Health Eating Index (HEI) has been demonstrated with plasma biomarkers [126,127], where a higher score in the SHEI was associated with blood concentrations of certain markers with a protective effect against particular diseases. Finally, this study included people aged ≥ 65 years old, and the total sample was therefore representative of all adults living in Spain.

### 4.3. Implications for Research and Practice

The findings of this work offer useful insights for further studies. Regardless of mental status, a high percentage of people had a moderate diet quality, which suggests that more effort needs to be made to improve healthy dietary habits. In addition, our findings could help to identify which age groups had a poor and/or moderate diet quality. In this case, it would be desirable for public health policies to promote healthy eating habits, especially among young people [128,129]. In particular, it is crucial to target favorable opinions of family, friends and health professionals in relation to the consumption of fruit and vegetables [130]. One way could be by promoting short cooking programs to improve young people’s skills and confidence in certain areas of food literacy, especially those related to the consumption of fruit and vegetables [131,132]. Although middle-aged adults and older individuals had higher diet quality index scores in comparison with other age groups, further intervention studies are required to promote healthy diet quality in these age groups, considering the psychological and social factors [133]. In addition, the results of the multivariate analysis may be used as a guide to improve Spanish public health policies and health promotion guidelines about diet quality [1]. Lastly, it would be of great interest to carry out further longitudinal studies to evaluate the impact of CMD on diet quality in the stages of life highlighted in this study.

## 5. Conclusions

Regardless of mental status, a better diet quality could be observed in older adults than emerging and young adults. In this regard, older adults with and without CMD meet the existing dietary guidelines in terms of daily consumption of bread/grains, fruit and dairy products. Nevertheless, a higher frequency of daily consumption of bread/grains is observed among middle-aged and older adults with CMD compared to those without CMD. In contrast, the consumption ‘never or hardly ever’ of soft drinks, sweets and cold meats and cuts increases with age among people with CMD in comparison to those without CMD, except in older adults. Among people with CMD, not having primary studies is considered a risk factor for having a poor or moderate diet quality. In contrast, in adults, overweight or obese and doing leisure-time physical activity are considered protective factors against having a poor or moderate diet quality. Among individuals without CMD, the likelihood of a having poor or moderate diet quality decreases in those who have university studies; however, it increases in those who are underweight or do not do any physical activity in their leisure time.

## Figures and Tables

**Table 1:** Sociodemographic, lifestyle, and health-related characteristics of participants (*n* = 60,624) (SNHS 2006, SNHS 2011/12 and SNHS 2017).

Variables	People without Common Mental Disorders *n* = 48,079 (%)	People with Common Mental Disorders *n* = 12,545 (%)
Gender		
Female	24,817 (75.12%)	8219 (24.88%)
Male	23,262 (84.32%)	4326 (15.68%)
Age groups (years)		
Emerging adults	2881 (84.39%)	533 (15.61%)
Young adults	17,343 (82.06%)	3791 (17.94%)
Middle-aged adults	16,368 (78.58%)	4463 (21.42%)
Older adults	11,487 (75.35%)	3758 (24.65%)
Marital status		
Never-married	12,441 (81.69%)	2789 (18.31%)
Married	28,453 (81.23%)	6575 (18.77%)
Widowed	4277 (67.71%)	2040 (32.29%)
Separated/divorced	2908 (71.82%)	1141 (28.18%)
Social class		
Classes I and II	9909 (84.36%)	1837 (15.64%)
Classes III and IV	20,250 (79.85%)	5111 (20.15%)
Classes V and VI	17,920 (76.20%)	5597 (23.80%)
Educational level		
Without formal education	4637 (69.15%)	2069 (30.85%)
Completed primary studies	10,608 (77.00%)	3169 (23.00%)
Completed secondary studies or professional training	23,695 (80.43%)	5766 (19.57%)
University studies	9139 (85.57%)	1541 (14.43%)
Nationality		
Spanish	44,928 (79.27%)	11,751 (20.73%)
Foreign	3151 (79.87%)	794 (20.13%)
Population of town/city		
<10,000 inhabitants	11,901 (80.52%)	2879 (19.48%)
≥10,000 inhabitants	36,178 (78.92%)	9666 (21.08%)
Body Mass Index		
Underweight	800 (77.07%)	238 (22.93%)
Normal-weight	20,875 (80.39%)	5092 (19.61%)
Overweight	18,371 (80.01%)	4589 (19.99%)
Obese	8033 (75.36%)	2626 (24.64%)
Self-perceived health status		
Very good	9452 (92.64%)	751 (7.36%)
Good	26,519 (87.25%)	3877 (12.75%)
Fair	9934 (67.86%)	4704 (32.14%)
Poor	1832 (43.48%)	2381 (56.52%)
Very poor	342 (29.13%)	832 (70.87%)
Current smokers		
Yes	12,903 (78.68%)	3496 (21.32%)
No	35,176 (79.54%)	9049 (20.46%)
Alcohol consumption in the past twelve months		
Yes	20,943 (82.66%)	4394 (17.34%)
No	27,136 (76.90%)	8151 (23.10%)
Physical exercise in main activity		
Yes	31,838 (81.75%)	7108 (18.25%)
No	16,241 (74.92%)	5437 (25.08%)
Leisure-time physical activity		
Yes	30,527 (83.04%)	6233 (16.96%)
No	17,552 (73.55%)	6312 (26.45%)

**Table 2:** Frequency of food consumption and diet quality according to mental status among adults from all three survey periods (*n* = 60,624) (SNHS 2006, SNHS 2011/2012 and SNHS 2017).

Variables	People without Common Mental Disorders *n* = 48,079 (%)	People with Common Mental Disorders *n* = 12,545 (%)	*p*-Value
Frequency of bread/grains consumption			
Never or hardly ever	859 (1.79%)	329 (2.62%)	<0.001
<1 a week	663 (1.38%)	248 (1.98%)	
1–2 a week	1575 (3.27%)	440 (3.51%)	
≥3 times a week, but not daily	3044 (6.33%)	816 (6.50%)	
Daily	41,938 (87.23%)	10,712 (85.39%)	
Frequency of vegetable consumption			
Never or hardly ever	582 (1.21%)	227 (1.81%)	<0.001
<1 a week	1160 (2.41%)	358 (2.85%)	
1–2 a week	6318 (13.14%)	1714 (13.66%)	
≥3 times a week, but not daily	18,491 (38.46%)	4335 (34.56%)	
Daily	21,528 (44.78%)	5911 (47.12%)	
Frequency of fruit consumption			
Never or hardly ever	1462 (3.04%)	549 (4.38%)	<0.001
<1 a week	1434 (2.98%)	463 (3.69%)	
1–2 a week	4140 (8.61%)	1166 (9.29%)	
≥3 times a week, but not daily	8131 (16.91%)	1883 (15.01%)	
Daily	32,912 (68.46%)	8484 (67.63%)	
Frequency of consumption of dairy products			
Never or hardly ever	1072 (2.23%)	373 (2.97%)	<0.001
<1 a week	684 (1.42%)	185 (1.48%)	
1–2 a week	1389 (2.89%)	417 (3.32%)	
≥3 times a week, but not daily	3012 (6.26%)	751 (5.99%)	
Daily	41,922 (87.20%)	10,819 (86.24%)	
Frequency of meat consumption			
Never or hardly ever	580 (1.21%)	254 (2.03%)	<0.001
<1 a week	1107 (2.30%)	483 (3.85%)	
1–2 a week	13,089 (27.22%)	3942 (31.42%)	
≥3 times a week, but not daily	27,718 (57.65%)	6496 (51.78%)	
Daily	5585 (11.62%)	1370 (10.92%)	
Frequency of consumption of legumes			
Never or hardly ever	1163 (2.42%)	455 (3.63%)	<0.001
<1 a week	5200 (10.81%)	1452 (11.57%)	
1–2 a week	29,361 (61.07%)	7233 (57.66%)	
≥3 times a week, but not daily	11,515 (23.95%)	3132 (24.97%)	
Daily	840 (1.75%)	273 (2.17%)	
Frequency of consumption of cold meats and cuts			
Never or hardly ever	6314 (13.13%)	2387 (19.03%)	<0.001
<1 a week	8871 (18.45%)	2612 (20.82%)	
1–2 a week	14,190 (29.52%)	3515 (28.02%)	
≥3 times a week, but not daily	12,002 (24.96%)	2480 (19.77%)	
Daily	6702 (13.94%)	1551 (12.36%)	
Frequency of consumption of sweets			
Never or hardly ever	8181 (17.01%)	2626 (20.93%)	<0.001
<1 a week	7905 (16.44%)	2094 (16.69%)	
1–2 a week	9663 (20.10%)	2220 (17.70%)	
≥3 times a week, but not daily	7542 (15.69%)	1699 (13.54%)	
Daily	14,788 (30.76%)	3906 (31.14%)	
Frequency of consumption of soft drinks with sugar			
Never or hardly ever	23,519 (48.92%)	6945 (55.36%)	<0.001
<1 a week	8676 (18.05%)	1894 (15.10%)	
1–2 a week	6940 (14.43%)	1387 (11.05%)	
≥3 times a week, but not daily	3832 (7.97%)	883 (7.04%)	
Daily	5112 (10.63%)	1436 (11.45%)	
Diet quality			
Poor diet quality	1130 (2.35%)	354 (2.82%)	<0.001
Moderate diet quality	30,878 (64.22%)	7639 (60.89%)	
Good diet quality	16,071 (33.43%)	4552 (36.29%)	

**Table 3:** Overall diet quality score and food groups’ scores according to mental status of participants (*n* = 60,624) (SNHS 2006, SNHS 2011/12 and SNHS 2017).

Variables	People without Common Mental Disorders (*n* = 48,079) M (SD)	People with Common Mental Disorders (*n* = 12,545) M (SD)	*p*-Value
Bread/grains consumption (points)	9.40 (1.85)	9.25 (2.11)	<0.001
Vegetable consumption (points)	8.08 (2.14)	8.06 (2.28)	0.34
Fruit consumption (points)	8.62 (2.45)	8.45 (2.70)	<0.001
Consumption of dairy products (points)	9.37 (1.94)	9.28 (2.12)	<0.001
Meat consumption (points)	7.45 (2.33)	7.49 (2.46)	0.10
Consumption of legumes (points)	8.49 (2.31)	8.27 (2.51)	<0.001
Consumption of cold meats and cuts (points)	5.11 (3.08)	5.60 (3.17)	<0.001
Consumption of sweets (points)	4.53 (3.71)	4.76 (3.85)	<0.001
Consumption of soft drinks with sugar (points)	7.29 (3.36)	7.50 (3.44)	<0.001
Overall diet quality (points)	74.96 (10.86)	75.27 (11.64)	<0.01

M = mean; SD = standard deviation.

**Table 4:** Frequency of consumption recommended by the Spanish Society of Community Nutrition according to age groups and mental status (*n* = 60,624) (SNHS 2006, SNHS 2011/12 and SNHS 2017).

Frequency of Consumption Recommended by the Spanish Society of Community Nutrition	Type of Food	Age Groups	People with Common Mental Disorders *n* = 12,545 (%)	People without Common Mental Disorders *n* = 48,079 (%)	*p*-Value
Daily consumption	Bread/grains	Emerging adults (18–24 years old)	401 (3.74%)	2384 (5.69%)	<0.001
		Young adults (25–44 years old)	3113 (29.06%)	14,683 (35.01%)	
		Middle-aged adults (45–64 years old)	3879 (36.21%)	14,477 (34.52%)	
		Older adults (≥65 years old)	3319 (30.99%)	10,394 (24.78%)	
	Vegetables	Emerging adults (18–24 years old)	176 (2.98%)	895 (3.92%)	<0.001
		Young adults (25–44 years old)	1652 (27.95%)	6902 (32.06%)	
		Middle-aged adults (45–64 years old)	2250 (38.06%)	7913 (36.76%)	
		Older adults (≥65 years old)	1833 (31.01%)	5868 (27.26%)	
	Fruit	Emerging adults (18–24 years old)	220 (2.59%)	1287 (3.91%)	<0.001
		Young adults (25–44 years old)	2019 (23.80%)	10,198 (30.99%)	
		Middle-aged adults (45–64 years old)	3163 (37.28%)	11,985 (36.41%)	
		Older adults (≥65 years old)	3082 (36.33%)	9442 (28.69%)	
	Dairy products	Emerging adults (18–24 years old)	440 (4.06%)	2453 (5.85%)	<0.001
		Young adults (25–44 years old)	3199 (29.57%)	15,036 (35.87%)	
		Middle-aged adults (45–64 years old)	3797 (35.10%)	14,180 (33.82%)	
		Older adults (≥65 years old)	3383 (31.27%)	10,253 (24.46%)	
1–2 times a week	Meat	Emerging adults (18–24 years old)	111 (2.82%)	559 (4.27%)	<0.001
		Young adults (25–44 years old)	869 (22.04%)	3746 (28.62%)	
		Middle-aged adults (45–64 years old)	1500 (38.05%)	4714 (36.02%)	
		Older adults (≥65 years old)	1462 (37.09%)	4070 (31.09%)	
	Legumes	Emerging adults (18–24 years old)	260 (3.59%)	1685 (5.74%)	<0.001
		Young adults (25–44 years old)	2169 (29.99%)	10,524 (35.84%)	
		Middle-aged adults (45–64 years old)	2674 (36.97%)	10,211 (34.78%)	
		Older adults (≥65 years old)	2130 (29.45%)	6941 (23.64%)	
Never or hardly ever	Cold meats and cuts	Emerging adults (18–24 years old)	60 (2.51%)	235 (3.72%)	<0.001
		Young adults (25–44 years old)	526 (22.04%)	1812 (28.70%)	
		Middle-aged adults (45–64 years old)	771 (32.30%)	2049 (32.45%)	
		Older adults (≥65 years old)	1030 (43.15%)	2218 (35.13%)	
	Sweets	Emerging adults (18–24 years old)	61 (2.32%)	254 (3.11%)	<0.001
		Young adults (25–44 years old)	644 (24.52%)	2318 (28.33%)	
		Middle-aged adults (45–64 years old)	1007 (38.35%)	3165 (38.69%)	
		Older adults (≥65 years old)	914 (34.81%)	2444 (29.87%)	
	Soft drinks with sugar	Emerging adults (18–24 years old)	123 (1.77%)	517 (2.20%)	<0.001
		Young adults(25–44 years old)	1384 (19.93%)	5904 (25.10%)	
		Middle-aged adults(45–64 years old)	2604 (37.49%)	9056 (38.51%)	
		Older adults(≥65 years old)	2834 (40.81%)	8042 (34.19%)	
**Diet Quality**		**Age Groups**	**People with Common Mental Disorders** ***n* = 12,545 (%)**	**People without Common Mental Disorders** ***n* = 48,079 (%)**	***p*-Value**
Poor diet quality		Emerging adults (18–24 years old)	71 (20.06%)	299 (26.46%)	0.04
		Young adults (25–44 years old)	206 (58.19%)	636 (56.28%)	
		Middle-aged adults (45–64 years old)	61 (17.23%)	161 (14.25%)	
		Older adults (≥65 years old)	16 (4.52%)	34 (3.01%)	
Moderate diet quality		Emerging adults (18–24 years old)	416 (5.45%)	2265 (7.34%)	<0.001
		Young adults (25–44 years old)	2768 (36.24%)	13,019 (42.16%)	
		Middle-aged adults (45–64 years old)	2637 (34.52%)	10,032 (32.49%)	
		Older adults (≥65 years old)	1818 (23.80%)	5562 (18.01%)	
Good diet quality		Emerging adults (18–24 years old)	46 (1.01%)	317 (1.97%)	<0.001
		Young adults (25–44 years old)	817 (17.95%)	3688 (22.95%)	
		Middle-aged adults (45–64 years old)	1765 (38.77%)	6175 (38.42%)	
		Older adults (≥65 years old)	1924 (42.27%)	5891 (36.66%)	

**Table 5:** Association between diet quality and sociodemographic, lifestyle and health-related factors in people with common mental disorders (*n* = 12,545) (SNHS 2006, SNHS 2011/2012 and SNHS 2017).

Variables	Participants with Common Mental Disorders (*n* = 12,545)				
	Poor/Moderate Diet Quality (*n* = 7993)				
	*n* (%)	OR (CI 95%)	*p*-Value	ORa (CI 95%) ^1^	*p*-Value
Gender					
Female	3005 (37.60%)	0.68 (0.63–0.73)	<0.001	0.70 (0.65–0.77)	<0.001
Male	4988 (62.40%)	Reference		Reference	
Age groups (years)					
Emerging adults	487 (6.09%)	2.91 (2.13–3.97)	<0.001	2.44 (1.60–3.36)	<0.001
Young adults	2974 (37.21%)	Reference		Reference	
Middle-aged adults	2698 (33.75%)	0.42 (0.38–0.46)	<0.001	0.46 (0.41–0.51)	<0.001
Older adults	1834 (22.95%)	0.26 (0.24–0.29)	<0.001	0.31 (0.27–0.35)	<0.001
Marital status					
Never-married	2175 (27.21%)	2.31 (2.08–2.56)	<0.001	1.35 (1.21–1.52)	<0.001
Married	3982 (49.82%)	Reference		Reference	
Widowed	1035 (12.95%)	0.67 (0.61–0.74)	<0.001	1.09 (0.97–1.22)	0.13
Separated/divorced	801 (10.02%)	1.53 (1.34–1.76)	<0.001	1.36 (1.18–1.57)	<0.001
Social class					
Classes I and II	1144 (14.31%)	0.91 (0.82–1.02)	0.10		
Classes III and IV	3246 (40.61%)	0.96 (0.89–1.04)	0.35		
Classes V and VI	3603 (45.08%)	Reference			
Educational level					
Without formal education	1120 (14.01%)	0.55 (0.49–0.61)	<0.001	1.24 (1.09–1.40)	<0.01
Completed primary studies	1901 (23.78%)	0.70 (0.64–0.76)	<0.001	1.17 (1.06–1.29)	<0.01
Completed secondary studies or professional training	3939 (49.28%)	Reference		Reference	
University studies	1033 (12.93%)	0.94 (0.84–1.06)	0.34	0.91 (0.80–1.04)	0.16
Nationality					
Spanish	7353 (91.99%)	Reference		Reference	
Foreign	640 (8.01%)	2.49 (2.07–2.98)	<0.001	157 (1.30–1.90)	<0.001
Population of town/city					
<10,000 inhabitants	1794 (22.44%)	0.93 (0.85–1.01)	0.08		
≥10,000 inhabitants	6199 (77.56%)	Reference			
Body Mass Index					
Underweight	184 (2.30%)	1.53 (1.12–2.08)	<0.01	1.29 (0.93–1.79)	0.13
Normal-weight	3515 (43.98%)	Reference		Reference	
Overweight	2752 (34.43%)	0.67 (0.62–0.73)	<0.001	0.83 (0.76–0.91)	<0.001
Obese	1542 (19.29%)	0.64 (0.58–0.70)	<0.001	0.84 (0.76–0.94)	<0.01
Self-perceived health status					
Very good	532 (6.66%)	1.50 (1.27–1.77)	<0.001		
Good	2650 (33.15%)	1.33 (1.22–1.46)	<0.001		
Fair	2911 (36.42%)	Reference			
Poor	1391 (17.40%)	0.87 (0.78–0.96)	<0.01		
Very poor	509 (6.37%)	0.97 (0.84–1.13)	0.70		
Current smokers					
Yes	2689 (33.64%)	2.35 (2.15–2.57)	<0.001	1.59 (1.44–1.75)	<0.001
No	5304 (66.36%)	Reference		Reference	
Alcohol consumption in the past twelve months					
Yes	2925 (36.59%)	1.21 (1.12–1.31)	<0.001		
No	5068 (63.41%)	Reference			
Physical exercise in main activity					
Yes	4602 (57.58%)	Reference			
No	3391 (42.42%)	0.90 (0.84–0.97)	<0.01		
Leisure-time physical activity					
Yes	3863 (48.33%)	0.86 (0.80–0.93)	<0.001	0.78 (0.72–0.84)	<0.001
No	4130 (51.67%)	Reference		Reference	

^1^ OR = odds ratio; ORa = odds ratio adjusted for all sociodemographic, lifestyle and health-related factors; CI 95% = confidence Interval; *n* = number of people with a poor or moderate diet quality; Hosmer–Lemeshow test χ^2^ = 12.22, *p* = 0.14; Nagelkerke’s R^2^: 0.14; *p*-value < 0.001.

**Table 6:** Association between diet quality and sociodemographic, lifestyle and health-related factors in people without common mental disorders (*n* = 48,079) (SNHS 2006, SNHS 2011/2012 and SNHS 2017).

Variables	Participants without Common Mental Disorders (*n* = 48,079)				
	Poor/Moderate Diet Quality (*n* = 32,008)				
	*n* (%)	OR (CI 95%)	*p*-Value	ORa (CI 95%) ^1^	*p*-Value
Gender					
Female	15,288 (47.76%)	0.63 (0.60–0.65)	<0.001	0.62 (0.59–0.65)	<0.001
Male	16,720 (52.24%)	Reference		Reference	
Age groups (years)					
Emerging adults	2564 (8.01%)	2.19 (1.93–2.47)	<0.001	1.77 (1.56–2.01)	<0.001
Young adults	13,655 (42.66%)	Reference		Reference	
Middle-aged adults	10,193 (31.85%)	0.45 (0.43–0.47)	<0.001	0.48 (0.45–0.50)	<0.001
Older adults	5596 (17.48%)	0.26 (0.24–0.27)	<0.001	0.29 (0.26–0.31)	<0.001
Marital status					
Never-married	9765 (30.51%)	2.07 (1.97–2.18)	<0.001	1.31 (1024–1.38)	<0.001
Married	18,144 (56.68%)	Reference		Reference	
Widowed	2099 (6.56%)	0.55 (0.51–0.58)	<0.001	1.06 (0.99–1.14)	0.11
Separated/divorced	2000 (6.25%)	1.25 (1.15–1.36)	<0.001	1.20 (1.10–1.31)	<0.001
Social class					
Classes I and II	6309 (19.71%)	0.89 (0.84–0.93)	<0.001	0.89 (0.84–0.94)	<0.001
Classes III and IV	13,452 (42.03%)	Reference		Reference	
Classes V and VI	12,247 (38.26%)	1.09 (1.05–1.14)	<0.001	1.08 (1.03–1.13)	<0.01
Educational level					
Without formal education	2543 (7.95%)	0.49 (0.46–0.52)	<0.001	1.00 (0.93–1.08)	0.99
Completed primary studies	6473 (20.22%)	0.63 (0.60–0.66)	<0.001	1.02 (0.96–1.08)	0.53
Completed secondary studies or professional training	16,933 (52.90%)	Reference		Reference	
University studies	6059 (18.93%)	0.79 (0.75–0.83)	<0.001	0.90 (0.84–0.95)	<0.01
Nationality					
Spanish	29,539 (92.29%)	Reference		Reference	
Foreign	2469 (7.71%)	1.89 (1.73–2.06)	<0.001	1.23 (1.12–1.34)	<0.001
Population of town/city					
<10,000 inhabitants	7933 (24,78%)	1.01 (0.96–1.10)	0.82		
≥10,000 inhabitants	24,075 (75.22%)	Reference			
Body Mass Index					
Underweight	632 (1.97%)	1.69 (1.42–2.01)	<0.001	1.47 (1.23–1.77)	<0.001
Normal-weight	14,397 (44.98%)	Reference		Reference	
Overweight	11,894 (37.16%)	0.83 (0.79–0.86)	<0.001	0.98 (0.93–1.02)	0.55
Obese	5085 (15.89%)	0.78 (0.74–0.82)	<0.001	0.98 (0.93–1.04)	0.32
Self-perceived health status					
Very good	6876 (21.48%)	1.27 (1.20–1.33)	<0.001	1.02 (0.96–1.07)	0.61
Good	17,988 (56.20%)	Reference		Reference	
Fair	5910 (18.47%)	0.70 (0.66–0.73)	<0.001	0.93 (0.88–0.98)	<0.01
Poor	1048 (3.27%)	0.63 (0.58–0.70)	<0.001	0.91 (0.82–1.03)	0.06
Very poor	186 (0.58%)	0.57 (0.46–0.70)	<0.001	0.80 (0.64–0.99)	0.04
Current smokers					
Yes	9952 (31.09%)	2.01 (1.92–2.10)	<0.001	1.42 (1.36–1.50)	<0.001
No	22,056 (68.91%)	Reference		Reference	
Alcohol consumption in the past twelve months					
Yes	14,321 (44.74%)	1.16 (1.11–1.20)	<0.001	1.04 (1.01–1.09)	0.04
No	17,687 (55.26%)	Reference		Reference	
Physical activity in main activity					
Physically active	21,164 (66.12%)	Reference			
Not physically active	10,844 (33.88%)	1.01 (0.97–1.06)	0.52		
Leisure-time physical activity					
Yes	19,381 (60.55%)	Reference		Reference	
No	12,627 (39.45%)	1.47 (1.42–1.53)	<0.001	1.47 (1.40–1.53)	<0.001

^1^ OR = odds ratio; Ora = odds ratio adjusted for all sociodemographic, lifestyle and health-related factors; CI 95% = confidence interval; *n* = number of people with a poor or moderate diet quality; Hosmer–Lemeshow test χ^2^ = 19.53, *p* = 0.12; Nagelkerke’s R^2^: 0.14; *p*-value < 0.001.

## Data Availability

The data are available as Supplementary Material (File S1: research data).

## Supplementary Materials

The following are available online at https://www.mdpi.com/article/10.3390/nu13051727/s1, Table S1: criteria to define the score for each item of the Spanish Health Eating Index (SHEI), File S1: research data.
