# Supplementary material for: Diet Quality According to Mental Status and Associated Factors during Adulthood in Spain
Source: Nutrients. 2021 May 19;13(5):1727. doi: 10.3390/nu13051727 (PMC8160880; doi:10.3390/nu13051727)
Supplement: Supplementary file 1 [file nutrients-13-01727-s001.zip › nutrients-1129199-supplementary.pdf]

nutrients

MDPI

Supplementary

Diet Quality According to Mental Status and Associated Factors during Adulthood in Spain

Jesús Cebrino [1] and Silvia Portero de la Cruz [2,*]

Supplementary Table S1. Criteria to define the score for each item of the Spanish Health Eating Index (SHEI).

Criteria to define the score for each item of the Spanish Health Eating Index (SHEI)					
**Variables**	**Criteria for a maximum score of 10**	**Criteria for a maximum score of 7.5**	**Criteria for a maximum score of 5**	**Criteria for a maximum score of 2.5**	**Criteria for a maximum score of 0**
Daily					
Bread/grains	Daily	≥ 3 times a week, but not daily	1–2 a week	< 1 a week	Never or almost never
Vegetables	Daily	≥ 3 times a week, but not daily	1–2 a week	< 1 a week	Never or almost never
Fruit	Daily	≥ 3 times a week, but not daily	1–2 a week	< 1 a week	Never or almost never
Dairy products	Daily	≥ 3 times a week, but not daily	1–2 a week	< 1 a week	Never or almost never
Weekly consumption					
Meat	1–2 a week	≥ 3 times a week, but not daily	< 1 a week	Daily	Never or almost never
Legumes	1–2 a week	≥ 3 times a week, but not daily	< 1 a week	Daily	Never or almost never
Occasional consumption					
Cold meats and cuts	Never or almost never	< 1 a week	1–2 a week	≥ 3 times a week, but not daily	Daily
Sweets	Never or almost never	< 1 a week	1–2 a week	≥ 3 times a week, but not daily	Daily
Soft drinks with sugar	Never or almost never	< 1 a week	1–2 a week	≥ 3 times a week, but not daily	Daily
Variety	2 points if participant achieve each of the daily recommendations, 1 point if participant achieve each of the weekly recommendations.				

Each item scored from 0 to 10 depending to the criteria of the Spanish Health Eating Index (SHEI) [1], which 10 points means that the recommendations proposed by the Spanish Society of Community Nutrition (SSCN) [2].

Supplementary references

1. Norte Navarro, A.; Ortiz Moncada, R. Spanish diet quality according to the healthy eating index. *Nutr. Hosp*. **2011**, *26*, 330–336, doi: 10.1590/S0212-16112011000200014.
2. Spanish Society of Community Nutrition (SSCN). Healthy dietary guidelines (2014). Available online: http://www.nutricioncomunitaria.org/es/otras-publicaciones (accessed on 13 December 2020).
